# *miR-205* Expression Promotes Cell Proliferation and Migration of Human Cervical Cancer Cells

**DOI:** 10.1371/journal.pone.0046990

**Published:** 2012-10-03

**Authors:** Hong Xie, Yungang Zhao, Stefano Caramuta, Catharina Larsson, Weng-Onn Lui

**Affiliations:** 1 Department of Molecular Medicine and Surgery, Karolinska Institutet, Cancer Center Karolinska, Stockholm, Sweden; 2 Tianjin Key Laboratory of Exercise Physiology and Sports Medicine, Tianjin Sport University, Tianjin, China; University of Hong Kong, Hong Kong

## Abstract

MicroRNAs (miRNAs) are short non-coding RNA regulators that control gene expression mainly through post-transcriptional silencing. We previously identified *miR-205* in a signature for human cervical cancer using a deep sequencing approach. In this study, we confirmed that *miR-205* expression was frequently higher in human cervical cancer than their matched normal tissue samples. Functionally, we demonstrate that *miR-205* promotes cell proliferation and migration in human cervical cancer cells. To further understand the biological roles of *miR-205*, we performed *in vivo* crosslinking and Argonaute 2 immunoprecipitation of miRNA ribonucleoprotein complexes followed by microarray analysis (CLIP-Chip) to identify its potential mRNA targets. Applying CLIP-Chip on gain- and loss-of-function experiments, we identified a set of transcripts as potential targets of *miR-205*. Several targets are functionally involved in cellular proliferation and migration. Two of them, CYR61 and CTGF, were further validated by Western blot analysis and quantification of mRNA enrichment in the Ago2 immunoprecipitates using qRT-PCR. Furthermore, both *CYR61* and *CTGF* were downregulated in cervical cancer tissues. In summary, our findings reveal novel functional roles and targets of *miR-205* in human cervical cancer, which may provide new insights about its role in cervical carcinogenesis and its potential value for clinical diagnosis.

## Introduction

Cervical cancer, the third most common cancer among women worldwide [1], is strongly associated with infection and subsequent transformation of cervical cells by specific human papillomavirus (HPV) subtypes [2]. The fact that cervical cancer develops from well-recognized pre-malignant forms, offers an important opportunity for early diagnosis and prevention. Today such primary screening includes cytological analyses and HPV identification. However, these examinations cannot reliably distinguish the lesions with invasive potential from the lesions that will spontaneously regress. Therefore, development of more robust markers for disease progression would be valuable supplements to the current screening methods.

MicroRNAs (miRNAs) are short non-coding RNAs (∼22-nucleotides) that generally control gene expression at the post-transcriptional level through mRNA degradation and/or translational repression [3]. These tiny molecules have been demonstrated to play important roles in a broad range of physiological and pathological processes, including cancer development and progression. We, and others, have previously identified altered miRNA expression signatures in human cervical cancer [4]–[10]. Several of these miRNAs have consistently been reported as dysregulated in cervical cancer (*e.g*. *miR-143*, *miR-145*, *miR-21* and *miR-205*). A few have also been functionally characterized in human cervical cancer cells. Among them, *miR-143*, *miR-145* and *miR-34a* have been shown to inhibit cell proliferation, and *miR-146a* and *miR-21* to increase cell growth [8], [10], [11]. *miR-23b* was recently found to repress the expression of urokinase-type plasminogen activator (uPA) and induce cell migration in human cervical cancer cells [12]. Taken together, these observations suggest that dysregulated miRNAs have a functional role in cervical cancer development and may become applied as diagnostic tools.

In this study, we examined the functional role of *miR-205* in human cervical cancer. This miRNA was one of the most significant miRNAs used for cervical cancer class prediction and was significantly overexpressed in cervical cancer samples compared to matched normal counterparts [9]. Increased expression of *miR-205* has also been observed in endometrial adenocarcinoma [13], head and neck squamous cell carcinoma cell lines [14], squamous cell lung carcinoma [15] and ovarian cancer [16]. By contrast, reduced expression of *miR-205* has been reported in melanoma [17] and cancers of the esophagus [18], kidney [19], bladder [20], [21], breast [22], and prostate [23].

Based on the above studies, *miR-205* may function as an oncogene or tumor suppressor gene depending on the cellular contexts. Consistent with its dual role, several studies have demonstrated its tumor promoting and suppressive roles in different cancer cell lines. For examples, *miR-205* has been shown to suppress cell migration/invasion through epithelial-to-mesenchymal transition in both human prostate and breast cancer cells [23], [24], as well as to target *HER3* tyrosine kinase receptor in breast cancer cells [22]. In support of an oncogenic function, *miR-205* was found to target *SHIP2* for Akt survival signaling in head and neck squamous cell carcinoma cells [14]. Given the complexity of its functionality, it would be of interest to investigate the functional roles of *miR-205* in cervical cancer development.

Here we describe the functional consequences of *miR-205* regulation in human cervical cancer cells. In gain- and loss-of-function experiments, we demonstrate that *miR-205* regulates cell proliferation and migration in human cervical cancer cells. We further identified a set of putative *miR-205* targets using a biochemical approach. Several of these candidate targets are functionally associated with cell proliferation and migration. Two of the potential *miR-205* mRNA targets were further validated in cell culture experiments. Our findings provide an important lead for further insights into the functional role of *miR-205* in human cervical cancer development.

## Results

### *miR-205* Expression in Human Cervical Cancer Samples

**Figure 1:**
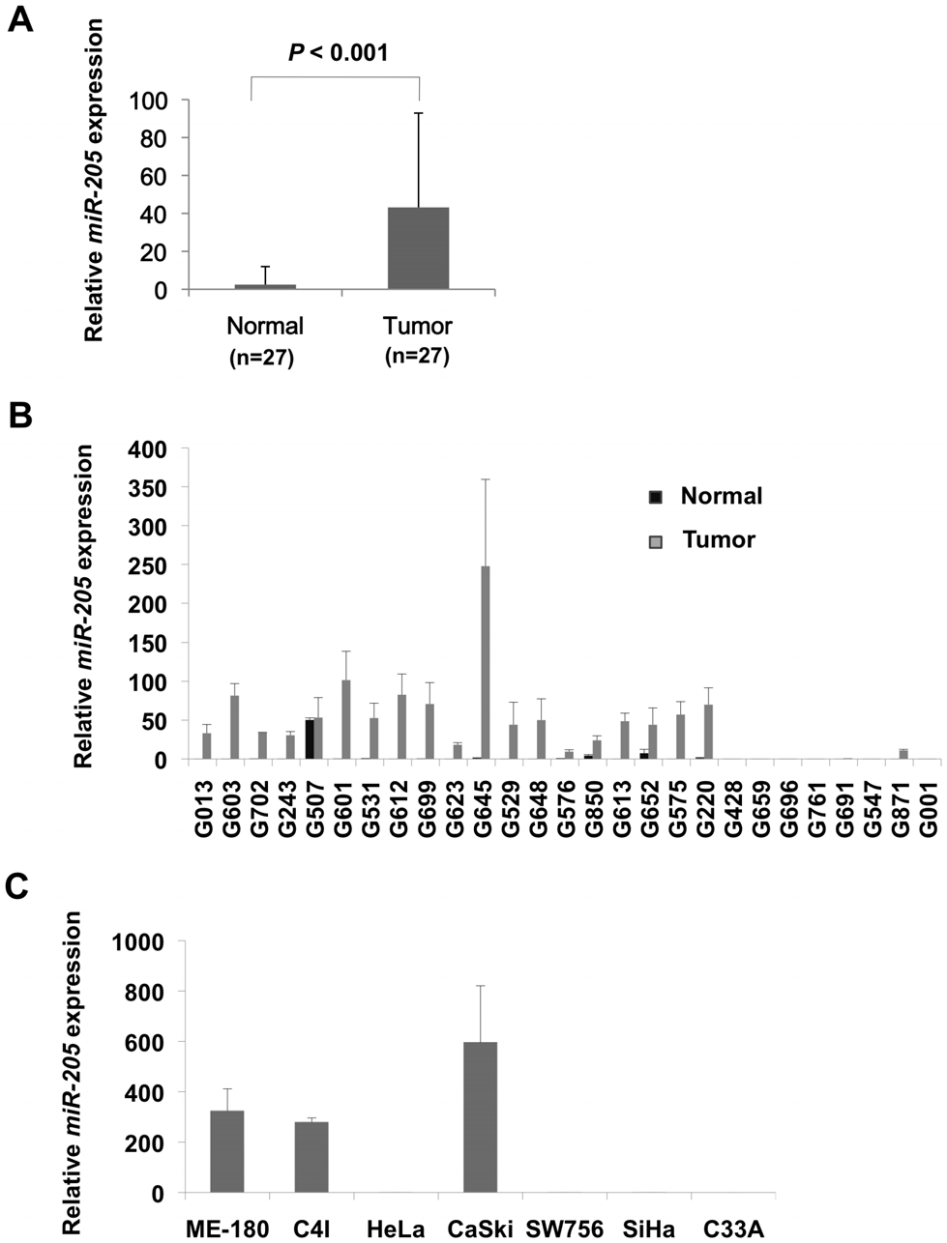
Real time quantitative RT-PCR of *miR-205* expression in human cervical tumors, normal cervices and cervical cancer cell lines, normalized to the geometric mean of *RNU6B* and *RNU43*. (A) *miR-205* expression was significantly higher in the tumors than the normal samples (*P*<0.001; paired t-test). (B) Relatively higher expression of *miR-205* was found in a majority of tumor samples as compared to their normal counterparts. (C) High expression of *miR-205* was detected in ME-180, C4I and CaSki cells, and low or undetectable expression level was found in HeLa, SW756, SiHa and C33A cells. Data presented represent mean of three independent experiments with triplicates. Error bars represent standard deviations from the mean.

We previously identified a set of miRNAs that could distinguish cervical cancer samples from their normal counterparts using a sequencing-based miRNA profiling approach [9]. In that classifier, *miR-205* had the highest score, suggesting an important function in cervical cancer development. To confirm the altered expression level of *miR-205* in cervical cancer, we measured *miR-205* expression by real-time quantitative reverse transcription-PCR (qRT-PCR) in 27 matched pairs of cervical cancer and normal tissue. In agreement with the sequencing-based results, *miR-205* was found significantly overexpressed in human cervical cancer as compared with their normal counterparts *P*<0.001; Figure 1A). In 19 cases (∼70%) the expression of *miR-205* was strongly increased in the tumor samples as compared to their normal counterparts; while in the remaining 8 cases, the cancer and normal samples exhibited low but comparable expression levels of *miR-205* (Figure 1B).

### Functional Consequences of *miR-205* Regulation in Human Cervical Cancer Cells

The functional consequences of altered *miR-205* expression were investigated in cervical cancer cell lines with high or low levels of endogenous *miR-205*. For this purpose *miR-205* was quantified in human cervical cancer cell lines by qRT-PCR. Among the 7 cell lines analyzed, *miR-205* was found highly expressed in ME-180, C4I and CaSki, while low expression/barely detectable levels were found in HeLa, SW756, SiHa and C33A (Figure 1C). Next we transfected CaSki cells with a miRNA inhibitor (Anti-miR-205), and HeLa and SW756 cells with a miRNA mimic (Pre-miR-205), and determined the effect of *miR-205* silencing or overexpression on cell proliferation, apoptosis and migration. As negative controls, we used a miRNA precursor or inhibitor without sequence homology to any human transcripts.

**Figure 2:**
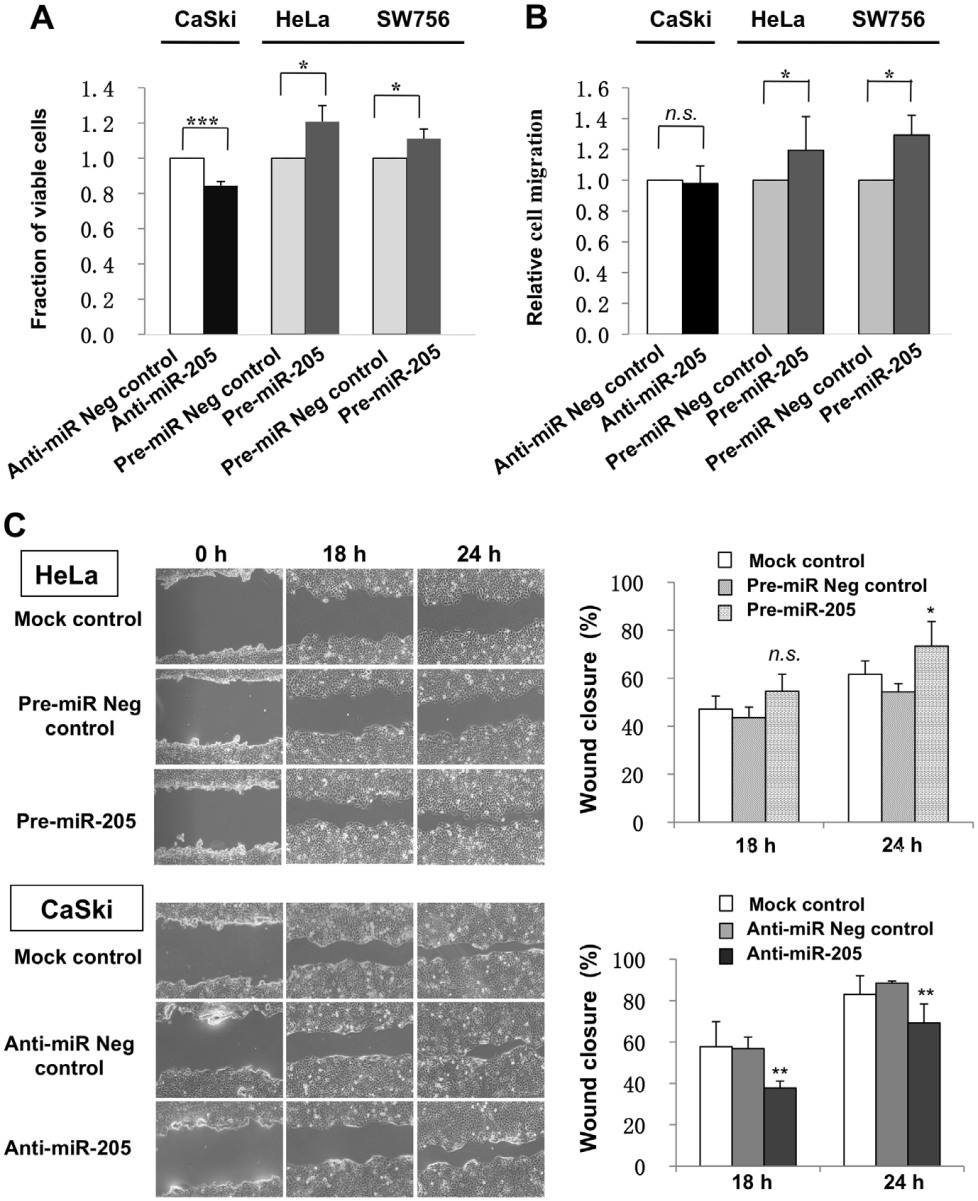
Functional analyses of *miR-205* regulation in cervical cancer cell lines. (A) Cell proliferation was assessed in human cervical cancer cell lines transfected with a *miR-205* mimic (Pre-miR-205), inhibitor (Anti-miR-205) or corresponding negative control (Anti-miR Neg control or Pre-miR Neg control) using WST-1 assay. Relative cell growth was normalized to its respective control-treated cells. (B) Graphs showing relative cell migration in both *miR-205* inhibition and overexpression experiments as evaluated by Transwell migration assay. (C) Representative images of cell migration evaluated by wound healing assay. Scratch wounds were made on confluent monolayer cultures after 48 h of transfection. Images of wound repair were taken at 0, 18 and 24 h after wound (left panel). The percentage of wound closure was normalized by wound area at 0 h (right panel). Data presented represent mean of three independent experiments. Error bars represent standard deviations from the mean. All comparisons were evaluated using t-test. *P<0.05; **P<0.01; ***P<0.001; *n.s.* = not significant.

We observed that inhibition of *miR-205* expression in CaSki cells led to a significant decrease in cell growth (∼15%; *P*<0.001), while overexpression of *miR-205* in HeLa and SW756 cells resulted in significant increases of cell proliferation (∼20% and ∼11%, respectively; *P*<0.05), as compared to the respective negative controls (Figure 2A). Taken together, both gain- and loss-of-function experiments consistently supported effects on cell proliferation.

Effects on cell migration were demonstrated using the Transwell and wound healing migration assays. Using the Transwell assay, we showed that cell migration was significantly enhanced by *miR-205* overexpression in both HeLa and SW756 cell lines (∼20% and ∼30%, respectively; *P*<0.05). However, *miR-205* suppression in CaSki cells did not lead to a significant decrease of cell migration (Figure 2B). The wound healing migration assay revealed that *miR-205* overexpression in HeLa cells enhanced the ability to close the wound compared with the Pre-miR negative control-treated and mock-transfected cells. Similarly, wound closure was retarded upon silencing of *miR-205* expression in CaSki cells (Figure 2C).

Treatment with *miR-205* mimic in HeLa or inhibitor in CaSki cells did not result in any significant change of apoptosis (Figure S1). In control experiments efficient transfection was demonstrated by significantly altered *miR-205* level, and significant induction of apoptosis was observed after camptothecin treatment (Figure S1).

### Identification of *miR-205* Target Genes

To further understand the biological function of *miR-205*, we identified *miR-205* targets using *in vivo* crosslinking and RNA immunoprecipitation coupled with microarray (CLIP-Chip). mRNAs bound to the miRNA machinery were purified by Argonaute 2 immunoprecipitation (Ago2 IP). The mRNA targets recovered from treated and control samples were differentially labeled with fluorescent dyes, and then hybridized to oligonucleotide microarrays to identify the mRNAs associated to microRNA ribonucleoprotein complex (miRNP). Here, we performed CLIP-Chip experiments for both *miR-205* overexpression in HeLa cells and inhibition in CaSki cells.

To verify the efficiency of Ago2 IP, we quantified the expression levels of *miR-21* and *miR-30a-5p* using qRT-PCR. These miRNAs were used as internal controls to evaluate the enrichment of miRNAs after CLIP because of their previously reported high expression levels in both HeLa and CaSki cells [5], [8]. We observed a significant enrichment of both *miR-21* (>100-fold, *P*<0.01) and *miR-30a-5p* (>10-fold, *P*<0.01) in anti-Ago2 IP compared to anti-IgG IP or input controls (Figure S2).

In our CLIP-Chip analysis, we excluded one of the replicate microarrays from the *miR-205* overexpression experiments due to poor hybridization signals. After filtering of background signals, we performed unsupervised hierarchical clustering of the five microarrays based on their mRNA expression patterns. We focused on the six clusters (including 270 transcripts/252 annotated genes) in which the expression patterns displayed enrichment in *miR-205* overexpression and depletion in *miR-205* suppression experiments (Figure S3 and Table S1). We performed functional annotation on the CLIP-Chip targets using GENECODIS program. Several functional groups were significantly enriched (*P*<0.05), including cell cycle, viral reproduction, DNA repair, apoptosis, cell proliferation and migration (Table 1). A detailed list of functional annotations is given in Table S2. Among the 75 candidate targets listed in Table 1, 71 were also predicted as *miR-205* targets in at least one prediction program (Table S3), and four targets (*BOD1*, *SEPT2*, *AAGAB* and *DCAF13*) were not predicted by any of the programs used in this study.

**Table 1:** Selected functional categories of *miR-205* targets obtained from CLIP-Chip experiments*.

Annotations (GO number)
*Genes included in category*
**Cell cycle** (GO:0000278, 0000084, 0000075, 0007049, 0000216, 0000082, 0006281)
*CCNB2, POLD3, NUP37, CDC20, SKP2, PSMD2, PSME2, PSMD7, AKAP9, PLK2, PSMD1, PSMD13,*
*RPA2, CDC23, AURKA, SEH1L, FEN1, NUSAP1, CHMP1B, BOD1, CSNK1A1, SEPT2, SUPT16H,*
*DDB1, RBX1, POLR2B, UBE2T, BCCIP, UBE2D3, TP53BP1, RAD51, TDG, RAD51C*
**Cell proliferation** (GO:0008283, 0001558)
*SKP2, KRT16, DUSP22, CDV3, YAP1, CYR61, CKLF, CTGF, FOXM1*
**Cell migration** (GO:0016477, 0030335)
*JUP, TNFAIP1, CTGF, PODXL, CYR61, MAP2K1*
**Apoptosis** (GO:0006915)
*BLCAP, HMGB2, HINT2, ECT2, DUSP22, PSMD2, TNFAIP1, PNMA1, UBE2D3, PSME2, PSMD7,*
*RRAGA, PSMD1, TIAL1, PSMD13*
**Viral reproduction** (GO:0016032)
*NUP37, RPL26L1, SUPT16H, RBX1, GTF2A2, POLR2B, PSMD2, PSME2, PSMD7, SLC25A4, PSMD1,*
*PSMD13, SEH1L*
**Translation** (GO:0006412)
*RPL26L1, EIF4E2, COPS5, EIF3E, MTIF2, MRPS33, MRP63, MRPL47, MRPS23, MRPS14*
**Protein transport** (GO:0015031)
*AAGAB, TIMM9, NUP37, SENP2, VPS37C, CHMP1B, RAB31, RAB35, C3orf31, CHMP2A, RAB1A, SEH1L*
**Protein ubiquitinylation** (GO:0016567)
*RBX1, RNF220, TNFAIP1, UBE2D3, DCAF13, CDC23*

* Functional annotations were performed using GENECODIS 2.0 (http://genecodis.decya.ucm.es/analysis/). A detailed list of all significant annotated functional groups is available in Table S2.

**Figure 3:**
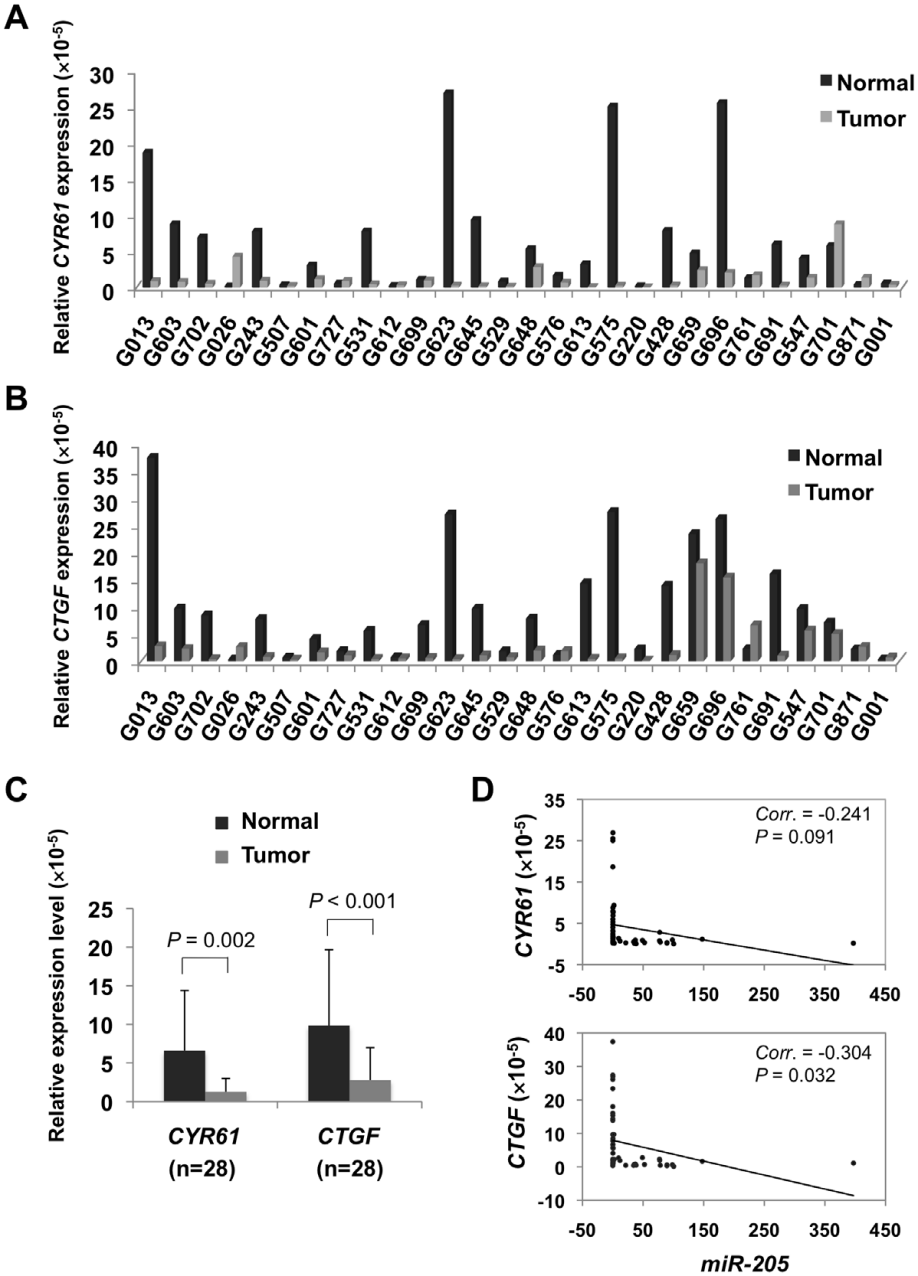
*CYR61* and *CTGF* mRNAs expression in human cervical samples, and their correlations with *miR-205* expression. Relatively lower expression of *CYR61* (A) and *CTGF* (B) was found in a majority of tumor samples as compared to their normal counterparts (n = 28). (C) The expression of *CYR61* and *CTGF* was significantly lower in the tumors than the normal samples (*P* = 0.002 and *P*<0.001, respectively; paired t-test). (D) Inverse correlation between the expression level of *miR-205* and *CYR61* (upper) or *CTGF* (lower). The expression relationship was evaluated by Pearson’s correlation analysis. *P*<0.05 was considered statistically significant.

Among the candidate target genes, we found *CYR61* and *CTGF* were associated with both cell proliferation and migration (Table 1), and it is consistent with our functional consequences observed in this study. To further understand the expression relationship between *miR-205* and *CYR61* or *CTGF*, we determined the expression of *CYR61* and *CTGF* in 28 matched pairs of cervical cancer and normal tissues using qRT-PCR. Our results revealed significantly lower expression of both *CYR61* and *CTGF* in human cervical cancer samples as compared with their normal counterparts (*P* = 0.002 and *P*<0.001, respectively; Figure 3A–C). Interestingly, the expression patterns of these two selected genes were inversely correlated with the *miR-205* expression (*CYR61*, *Corr* = -0.241, *P* = 0.091; *CTGF*, *Corr* = -0.304, *P* = 0.032; Figure 3D). The observed inverse expression patterns, together with the predicted *miR-205* binding sites (Table S3, Figure S4), provide further evidence for *CYR61* and *CTGF* as *miR-205* targets.

### Validation of *CYR61* and *CTGF* as *miR-205* Target Genes

**Figure 4:**
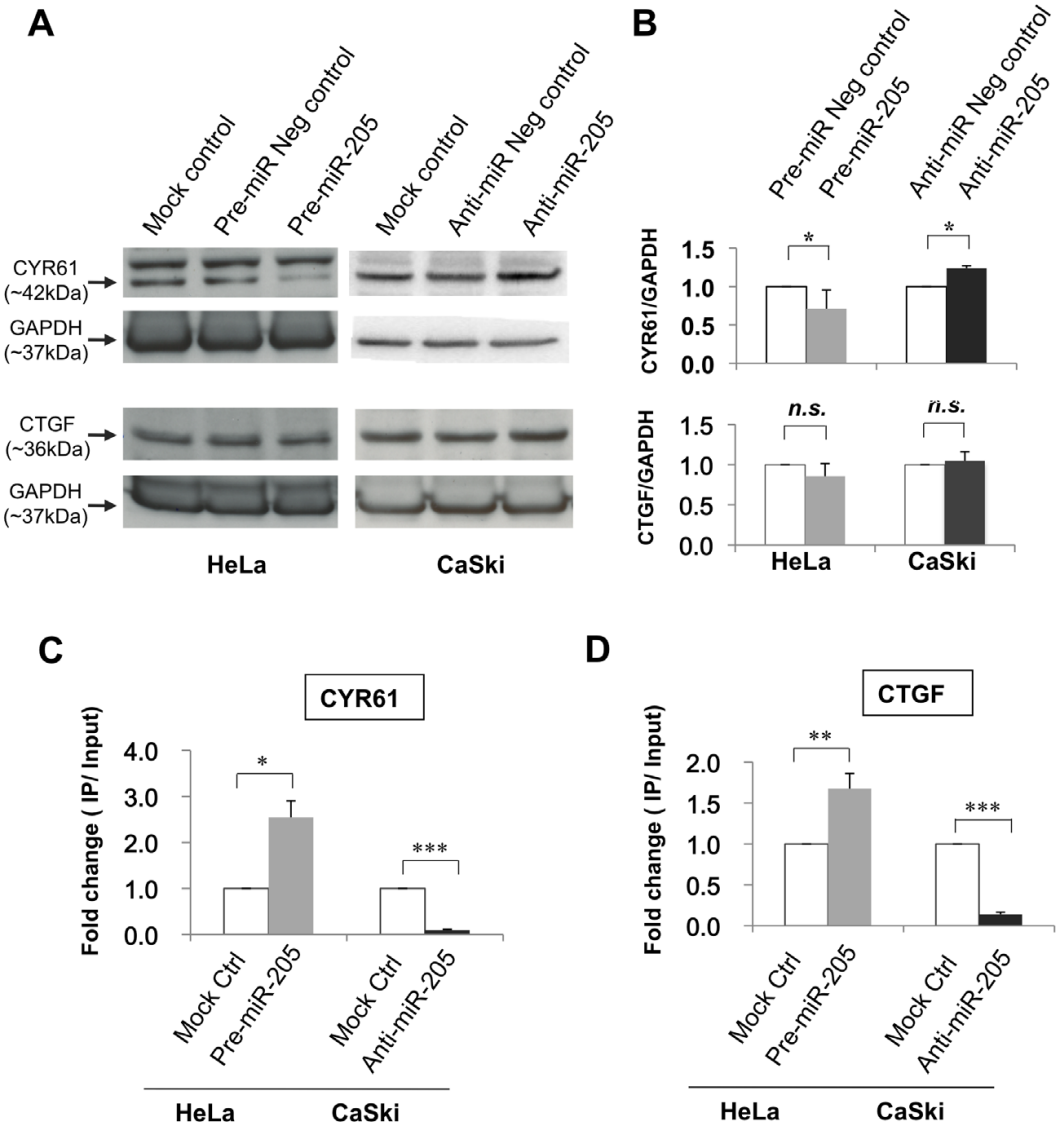
Evaluation of *CYR61* and *CTGF* as targets of *miR-205*. (A) Representative Western blot showing the protein expression levels of CYR61 and CTGF in cells transfected with a *miR-205* mimic, *miR-205* inhibitor, or corresponding scramble and mock transfection controls. (B) CYR61 protein expression was significantly repressed in *miR-205*-overexpressing (treated with Pre-miR-205) cells and significantly increased in *miR-205*-depleting (treated with Anti-miR-205) cells as compared to their respective negative controls. CTGF protein expression was slightly repressed in HeLa cells treated with Pre-miR-205, and slightly increased in CaSki cells treated with Anti-miR-205, but the effect was not statistically significant. Data presented represent mean of at least four independent experiments. qRT-PCR analysis of *CYR61* (C) and *CTGF* (D) mRNA in the Ago2-immunoprecipitated RNAs of *miR-205*-overexpressing or -depleted cells as compared to mock-transfection control. Relative expression level of individual mRNAs was normalized to *miR-21* expression (as endogenous control for Ago2 IP RNA). Fold change was calculated by dividing the normalized expression values of Ago2-immunoprecipiated samples by the normalized expression values of its respective input samples. Data presented represent mean of at least three independent experiments. Error bars represent standard deviations from the mean. All comparisons were evaluated using *t*-test. *P<0.05; **P<0.01; ***P<0.001; *n.s.* = not significant.

To determine if *CYR61* and *CTGF* could be targets of *miR-205* in human cervical cancer cells, we applied two different approaches. First, we evaluated the protein expression levels of CYR61 and CTGF in both *miR-205*-overexpressing and -depleted cervical cancer cells using Western blot analysis. As shown in Figure 4 (A and B), *miR-205* over-expression in HeLa cells resulted in a significant decrease in CYR61 protein expression (∼30%, *P* = 0.045). Inhibition of endogenous *miR-205* expression in CaSki cells significantly increased CYR61 protein level (∼15%, *P* = 0.016). For CTGF, we observed a slight decrease or increase (but not statistically significant) protein expression in *miR-205*-overexpressing or -depleted cells, respectively. A plausible explanation is that CTGF can be regulated by multiple miRNAs or factors, and modulating *miR-205* expression alone was not sufficient to yield significant changes on CTGF protein expression level.

In the second approach, we quantified *CYR61* and *CTGF* mRNAs in Ago2-immunoprecipitated mRNAs in both *miR-205*-overexpressing and depleted cells using qRT-PCR, and compared their levels with mock-transfected controls. This experiment is based on the assumption that miRNAs and their mRNA targets are physically associated with the Ago2-containing protein complex. Therefore, we expected enrichment of target mRNAs in *miR-205* over-expressing cells, or depletion of targets in cells with *miR-205* inhibition. Indeed, we observed significant enrichments of *CYR61* (*P* = 0.050) and *CTGF* (*P* = 0.007) mRNAs in HeLa cells with overexpression of *miR-205*, and depletions of both transcripts in CaSki cells with *miR-205* inhibition (*P*<0.001 for both targets; Figure 4C and 4D). These results suggest that both *CYR61* and *CTGF* are targets of *miR-205* in human cervical cancer cells.

## Discussion

Observations of increased or decreased expression of *miR-205* in different tumor types suggest that *miR-205* may have different functions in cancer development depending on the cell type involved. In line with this notion, previous studies have demonstrated its tumor suppression function in both breast and prostate cancer cells [22]–[24], and its tumor promotion function in head and neck squamous cell carcinoma cells [14]. In this work, we further investigated its functional consequences and targets in human cervical cancer cells.

### *miR-205* Regulates Cell Proliferation and Migration in Human Cervical Cancer Cells

Here, we first confirmed by qRT-PCR that *miR-205* is significantly overexpressed in cervical cancer samples as compared to their normal counterparts. The result is in agreement with our previous sequencing based findings [9], and with the microarray data reported by Wang et al. [8]. Given the observed increased expression of *miR-205* in cervical cancer tissues, we further investigated the functional consequences of *miR-205* regulation in human cervical cancer cells. In both *miR-205*-overexpressing cells (HeLa and SW756), we observed significant effects on cell proliferation and migration. Following *miR-205* inhibition in CaSki cells, proliferation was significantly decreased, however the effect on cell migration was only revealed in the wound healing assay but not in the Transwell migration assay. One possible reason for this discrepancy is that cell migration depends on different factors in the respective assays. The cell migration that occurs during wound healing is dependent on cell-matrix interaction. However, in the Transwell assay, cells are first prepared in single cells suspension, which will disrupt cell-cell and cell-matrix interactions. Furthermore, cell migration in the Transwell assay may depend on the chemotactic gradient, which is not available in the wound healing assay.

Effects on cell proliferation and migration similar to those observed here in human cervical cancer have also been reported in other cell types. For example, *miR-205* overexpression led to an increased cell proliferation in mouse mammary epithelial cell progenitors [25] and cell migration in human keratinocytes [26]. By contrast, increased expression of *miR-205* was found to suppress cell proliferation in melanoma [17] and breast cancer cells [27], as well as cell migration in a variety of cancer cell lines, including SK-LU-1 small cell lung cancer [28], U87 glioblastoma [28] and A498 renal cancer [19]. Taken together, these findings further support the dual function of *miR-205* as a tumor suppressor or an oncogene.

### *CYR61* and *CTGF* as Novel Targets of *miR-205*

Because of its functional complexity, we applied a biochemical approach (CLIP-Chip) to identify the *miR-205-*target interactions *in vivo*. Using this approach, we identified a set of *miR-205* targets from both gain- and loss-of-function experiments. Among these, several are functionally related to cell proliferation and migration; which is consistent with the functional consequences observed in this study. Two of the target genes, *CYR61* and *CTGF*, were further validated at protein and/or RNA levels. However, their precise interaction site(s) needs to be further determined by luciferase reporter assays. In this study, we did not perform CYR61 and CTGF inhibition in cervical cancer cells, it is possible that other direct target(s) contributes to the *miR-205*-mediated effects on cellular proliferation and migration. Yet, these genes had significantly lower expression in cervical cancer samples than their normal counterparts, suggesting that they may play an important role in cervical carcinogenesis.

CYR61 and CTGF proteins are members of the cysteine rich 61/connective tissue growth factor/nephroblastoma (CCN) family of growth regulators. These proteins play diverse roles in many cellular processes, including development, cell proliferation, adhesion, migration, angiogenesis and tumorigenesis [29]. CCNs are aberrantly expressed in a wide range of tumor types [29]. Interestingly, both CYR61 and CTGF can function as tumor suppressors or oncogenes depending on the cellular context (see examples below); which is similar to the dual function of *miR-205*.

In concordance to our observations, deregulation of CYR61 and CTGF has also been shown in several other studies. For example, *CYR61* expression is down-regulated in cervical cancer [30], lung cancer [31]–[33], endometrial cancer [34] and hepatocellular carcinoma [35]; however, its up-regulation has been reported in multiple tumor types, including osteosarcoma [36], glioma [37], [38], and breast cancer [39], [40]. Similar to CYR61, previous studies have revealed conflicting expression patterns of CTGF in different tumor types. For example, decreased CTGF expression has been reported in lung cancer [31], breast cancer [40], Wilm’s tumor [41] and ovarian cancer [42]; while increased expression was found in papillary thyroid cancer [43], colorectal cancer [44], head and neck squamous cell carcinoma [45], [46] and glioblastoma [47].

Interestingly, *miR-205*, CYR61 and CTGF are involved in common functional processes and pathways. Similar to the functional consequences of *miR-205* observed in this study, CYR61 has been shown to suppress cell growth in lung cancer [32] and hepatocellular cancer [35]. On the other hand, silencing of CYR61 suppresses cell proliferation and migration in glioma [37] and pancreatic cancer cells [48]. Loss of *miR-205* expression leads to induction of epithelial-to-mesenchymal transition (EMT) [23], [24], while silencing of CYR61 expression inhibits EMT [48]. Depletion of *miR-205* and CYR61 expression inhibits Akt signaling in keratinocytes, oral squamous cell carcinoma cells [14] and glioma cells [37]. Like CYR61 and *miR-205*, CTGF has also been demonstrated to play both oncogenic and suppressor roles in a wide range of cancer cell types [45], [47], [49]–[51], and it is also involved in both EMT [52], [53] and the Akt pathway [54]–[56]. Despite the numerous studies mentioned above, the roles of CYR61 and CTGF in human cervical cancer remain unclear. It will be of interest to determine the functional roles of these factors in cervical cancer and to evaluate their interactions with *miR-205* in different cancer types.

In summary, we report functional effects on tumor phenotypes and novel targets of *miR-205* in human cervical cancer cells. We show that *miR-205* plays an oncogenic role in human cervical cancer by promoting cell proliferation and migration. Furthermore, we identified a set of novel *miR-205* targets using a combination of biochemical and microarray approach. Among them, *CYR61* and *CTGF* were further verified at protein and/or RNA levels. Importantly, these two genes were downregulated in human cervical cancer samples. Our findings suggest that *miR-205* and its targets (*e.g.* CYR61 and CTGF) may play important roles in the pathogenesis of cervical cancer, and that *miR-205* (and its targets) may provide potential diagnostic values for cervical pathology.

## Materials and Methods

### Tissue Samples and Ethics Statement

Thirty pairs of snap-frozen cervical tumor and matched normal tissues from adjacent regions of 30 patients were provided by the Gynecologic Oncology Group Tissue Bank (Columbus, Ohio). Tumor and normal tissue samples had been verified as tumor or non-tumor by histopathological examination of hematoxylin and eosin-stained paraffin sections. Twenty-nine pairs of the samples were included in our previous small RNA profiling by deep sequencing technology [9]. The study was approved by Karolinska Institutet Ethics Committee. No written informed consent was needed because all clinical materials were deidentified. The ethic committee board of the Karolinska Institutet specifically waived the need for consent.

### Cervical Cancer Cell Lines

Seven human cervical cancer cell lines were used: CaSki, HeLa, SW756, ME-180, SiHa, C4I and C33A. CaSki and ME-180 cells were originally established from metastatic sites of cervical cancer, and the other cell lines were derived from primary cervical tumors [57]–[61]. These lines were kindly provided by Dr. Keng-Ling Wallin (Karolinska University Hospital, Sweden), and had been purchased from American Type Tissue Culture (ATCC). CaSki and ME-180 cells were grown in RPMI 1640, while HeLa, SW756, SiHa, C4I and C33A cells were cultured in DMEM medium. All cells were supplemented with 10% FBS and 1% penicillin/streptomycin (Invitrogen, Carlsbad, CA) and cultured at 37°C and 5% CO_2_ in a humidified incubator.

### TaqMan Quantitative Reverse Transcription-PCR (qRT-PCR)

Expression of mature miRNAs and mRNAs was quantified by qRT-PCR using an Applied Biosystems 7500 Fast Real-time PCR system (Applied Biosystems). RNA was extracted using mirVana miRNA isolation kit (Applied Biosystems/Ambion, Austin, TX), applying small RNA enrichment from tissue samples and total RNA isolation from cell lines. RNA concentrations were measured using a NanoDrop ND-1000 spectrophotometer (NanoDrop Technologies, Wilmington, DE).

For mature miRNA, cDNA was synthesized from 25 ng small RNA-enriched RNA for tissue samples, 120 ng total RNA for cell lines or 15 ng Ago2-immunoprecipitated RNAs using Taqman MicroRNA Reverse Transcription Kit (Applied Biosystems). Predesigned TaqMan MicroRNA Assays for *miR-205* (ID 000509), *miR-21* (ID 000397) and *miR-30a-5p* (ID 000417) were purchased from Applied Biosystems. All reactions were performed in triplicate on three independent occasions, and relative expression levels were normalized to the geometric mean of *RNU6B* (ID 001093) and *RNU43* (ID 001095), and reported as 2^−ΔCT^.

For mRNA quantification, cDNA was synthesized from 200 ng large RNA fraction (*i.e.* RNA fraction remained after small RNA enrichment) for tissue samples or 50 ng Ago2-immunoprecipitated RNAs using High Capacity cDNA Reverse Transcription kit (Applied Biosystems). qRT-PCR was performed for *CYR61* (Hs00155479_m1; Applied Biosystems) and *CTGF* (Hs00170014_m1; Applied Biosystems) mRNAs. All reactions were done in triplicate. For tissue samples, relative expression levels were normalized against *18S* (Hs99999901_s1; Applied Biosystems) and reported as 2^−ΔCT^. For quantification of mRNA enrichment in Ago2-immunoprecipitated RNAs, we used the endogenous *miR-21* for normalization due to the high abundance of *miR-21* in both CaSki and HeLa cells, and their direct association with Ago2 complexes. Furthermore, *miR-21* expression is not expected to be influenced in both *miR-205* overexpressing and depleted cells. To calculate the fold enrichment of individual target mRNA, the normalized expression level of target mRNA in the Ago2-immunoprecipitated RNAs was divided by its respective input RNA.

### *miR-205* Inhibition and Overexpression

For *miR-205* inhibition, CaSki cells were transiently transfected with 50 nM of Anti-miR-205 (Applied Biosystems/Ambion). As negative controls, the cells were transfected with mock reagent or Anti-miR Negative control #1 (Applied Biosystems/Ambion) in parallel. For *miR-205* overexpression, HeLa and SW756 cells were transfected with 10 nM Pre-miR-205, and Pre-miR Negative control #1 (Applied Biosystems/Ambion) or mock were used as negative controls. All cells were transfected with siPORT NeoFX transfection agent (Applied Biosystems/Ambion). Cells were collected 48–72 hours after transfection for subsequent experiments. Transfection efficiency was measured by quantification of the endogenous *miR-205* expression using qRT-PCR.

### Cell Proliferation Assay

Cell proliferation was measured using the WST-1 (4-[3-(4-iodophenyl)-2-(4-nitrophenyl)-2H-5-tetrazolio]-1,3-benzene disulfonate; 11644807001; Roche Applied Science, Mannheim, Germany) colorimetric assay. After 48 hours of transfection, 1×10^4^ cells/well (in 100 µl culture medium) were seeded into a 96-well plate and incubated for another 24 hours. Then, 10 µl of WST-1 reagent was added and incubated for 3 hours at 37°C. Absorbance was subsequently determined at wavelengths 450 nm (for measurements) and 650 nm (as reference) by a VERSAmax microplate reader (Molecular Devices, Sunnyvale, CA) and analyzed with SoftMax Pro 5 software (Molecular Devices). At least 8 replicate wells were included for each experimental group, and all experiments were repeated at least three times independently. Cell proliferation was calculated by subtracting the absorbance values of the samples from the media alone (background level). The relative cell proliferation was normalized by the respective control.

### Transwell Cell Migration Assay

BD Falcon™ 8.0-µm pore Transwell cell culture inserts (353097; BD Biosciences, Franklin lakes, NJ) were used to evaluate cell migration. The inserts were placed in a 24-well plate, containing 700 µl of medium with 10% FBS (lower chamber), for 30 minutes before seeding cells. After 48 hours of transfection, cells were harvested and counted by trypan blue staining in a TC10™ automated cell counter (Bio-Rad, Hercules, CA). 5×10^4^ cells/well (in 100 µl serum-free medium) were added to the upper chamber and incubated for 18 hours (HeLa and SW756 cells) or 48 hours (CaSki cells) at 37°C and 5% CO_2_. At the end of incubation, non-migrated cells on the top surface of membrane were removed using cotton swabs, followed by washing with PBS. Migrated cells on the bottom surface of membrane were fixed with 4% paraformaldehyde solution (19943, USB Corporation, Cleveland, OH) for 10 minutes, washed with PBS and stained with 0.5% crystal violet (prepared in 20% ethanol) for 10 minutes. The inserts were rinsed with tapped water and air-dried. For quantification of migrated cells, the stained cells were dissolved in 95% ethanol by gently shaking for 6 hours at room temperature. Absorbance was determined at 595 nm using a VERSAmax microplate reader (Molecular Devices). Cell migration was calculated by comparing the absorbance values of the samples after background subtraction and negative control-treated cells were used as negative controls. All the experiments were performed independently in triplicate.

### Wound Healing Assay

For wound-healing migration assay, cells (3.5×10^5^ in 2.5 ml/well) were transfected and seeded on 6-well plates. After 48 hours of transfection, a scratch wound was made on a confluent monolayer culture of HeLa and CaSki cells with a 100-µl-pipette tip and fresh media was added for further 24 hours incubation. The cells were imaged at three different time points (0 h, 18 h and 24 h) using an inverted microscopy system (Leica DM IL LED, Leica Microsystems GmbH, Wetzlar, Germany) equipped with ProgRes**®** MF camera (Jenoptik GmbH, Jena, Germany). All images were processed and quantified using Image J version 1.43 u (http://rsbweb.nih.gov/ij/). The percentage of wound closure (cell migration) was calculated as relative wound area at a given time point normalized by wound area at 0 h. All experiments were performed independently in triplicate.

### Apoptosis Assay

Apoptosis assay was performed using the caspase-3 colorimetric assay kit (K106-200; BioVision, Mountain View, CA) according to the manufacturer’s recommendations. In brief, 3×10^6^ transfected cells were harvested after 72 hours of transfection and re-suspended in 50 µl of chilled cell lysis buffer, followed by incubation on ice for 10 minutes. Protein lysates were quantified by BCA protein assay kit (23227; Pierce Biotechnology, Rockford, IL). 100 µg protein lysate was mixed with 50 µl of 2× Reaction Buffer and 5 µl of 4 mM caspase-3 substrate (DEVD-pNA), and incubated for 1 hour at 37°C. Detection of the chromophore p-nitroaniline (pNA) after cleavage from the labeled substrate DEVD-pNA was measured at 405 nm using a VERSAmax microplate reader (Molecular Devices) and analyzed with SoftMax Pro 5 software (Molecular Devices). Relative caspase-3 activity was determined by the absorbance values of the samples after background subtraction and compared with the respective negative control-treated cells. All experiments were replicated three times independently. As positive controls, HeLa and CaSki cells were treated with 100 µM camptothecin (an apoptosis inducer) for 15–18 hours.

### Argonaute 2 Immunoprecipitation (Ago2 IP)

After 72 hours of transfection, cells (from ten 10-cm tissue culture plates for each condition) were washed with cold PBS and irradiated for 120 mJ/cm^2^ in an UV cross-linker (UVC 500; Amersham Life Science, Arlington Heights, IL) for 30 seconds. Cell pellet was collected and then re-suspended in an equal volume (w/v) of lysis buffer [FNN0021; Invitrogen; supplemented with 1 mM Phenylmethanesulfonyl fluoride (PMSF, P7626; Sigma-Aldrich), 1 mM Dithiothreitol **(**DTT, 495714; Invitrogen), 1% Protease Inhibitor Cocktail (P8340; Sigma-Aldrich) and 200 U/ml RNaseOUT (10777-019; Invitrogen)], incubated for 10 minutes on ice, and lysed by vortexing. The cell lysate was stored at −80°C until use. After thawing on ice, the lysates were cleared by centrifugation at 14 000 rpm for 30 minutes at 4°C. To prepare antibody-coated beads, 120 µl of Protein G Sepharose 4 Fast Flow bead slurry (17-0618-01; GE Healthcare) was rinsed five times with 1 ml of NT2 buffer (50 mM Tris-HCl, pH 7.5, 150 mM NaCl, 1 mM MgCl_2_, 0.5% NP-40) and then incubated with 5 µg of mouse anti-human Ago2 (ab57113; Abcam, Cambridge, UK) or mouse IgG (I8765; Sigma-Aldrich) as isotype antibody control overnight at 4°C. The beads were then washed with cold NT2 buffer three times to remove the unbound antibodies. For immunoprecipitation, the cleared lysates were incubated with the antibody-coated Sepharose beads (in NT2 buffer supplemented with 1 mM DTT, 200 U/ml RNaseOUT, and 20 mM EDTA) overnight at 4°C on a rocker. The beads were washed three times with cold NT2 buffer for 10 minutes each at 4°C, followed by incubation with proteinase K (10 mg/ml) for 30 minutes at 55°C. Ago2-bound RNA was extracted with TRIzol reagent (Invitrogen).

### Microarray Experiments and Data Analysis

HEEBO oligonucleotide microarrays used in this study were produced by Stanford Functional Genomics Facility (http://www.microarray.org/sfgf/). The HEEBO microarrays contain ∼44,500 70-mer oligonucleotide probes, representing ∼30,000 unique transcripts. A detailed description of this probe set can be found at Stanford Functional Genomics Facility (http://www.microarray.org/sfgf/heebo.do).

RNAs obtained by Ago2 IP (∼250 ng of each sample) from six replicate experiments (Anti-miR-205 vs. mock control in CaSki cells and Pre-miR-205 vs. mock control in HeLa cells) were amplified using the Amino Allyl MessageAMP II aRNA kit (1753; Ambion). The amplified RNAs were fluorescently labeled by coupling to NHS-Cy3 (for Anti-miR-205 or Pre-miR-205 treated cells) or NHS-Cy5 (for mock transfected cells used as negative control). Samples were hybridized to the HEEBO microarrays at 65°C for 18–22 hours [62]. Arrays were stringently washed and immediately scanned using an Axon GenePix 4200A scanner (Molecular Devices). Images and fluorescence ratios were processed using GenePix Pro6.0 software (Molecular Devices), and data were uploaded into the Stanford Microarray Database (SMD; http://smd.stanford.edu/) for analysis.

To minimize errors, data were filtered to exclude measurements that did not have a regression correlation ≥0.6 between Cy3 and Cy5 signal, and intensity/background ratio ≥3 in at least one channel, for 80% of the arrays. Hierarchical clustering was performed with cluster 3.0 (http://bonsai.hgc.jp/~mdehoon/software/cluster/software.htm#ctv) and visualized with Java TreeView version 1.1.3 (http://jtreeview.sourceforge.net).

### Computational Analysis of *miR-205* Targets

Functional annotation of potential *miR-205* target genes obtained from CLIP-Chip data was performed using GENECODIS 2.0 (http://genecodis.dacya.ucm.es/analysis/). *miR-205* predicted targets were retrieved from miRecords (http://mirecords.biolead.org/) and the binding site predictions were performed using RNAhybrid (http://bibiserv.techfak.uni-bielefeld.de/rnahybrid/).

### Western Blot Analysis

Cells were collected after 72 hours post-transfection and lysed in NP-40 Cell Lysis Buffer (FNN0021; Invitrogen), with fresh addition of 1% Protease Inhibitor Cocktail (P8340; Sigma-Aldrich) and 1 mM PMSF (P7626; Sigma-Aldrich). After quantification with the BCA protein assay kit (23227; Pierce Biotechnology, Rockford, IL), 50 µg of whole cell lysate was separated in 10–20% Novex® Tricine gels (EC6625; Invitrogen) and transferred to nitrocellulose membranes (LC2001; Invitrogen). Novex Sharp Pre-stained Protein Standards (57318; Invitrogen) were used as molecular weight standards. Membranes were blocked with 5% non-fat milk in TBST (Tris-buffered saline/0.05% Tween 20), followed by incubating with CYR61 (1∶500 dilution; ab24448; Abcam) or CTGF (1∶5000 dilution; ab6992; Abcam) antibody overnight at 4°C. After washing for 3×10 minutes with TBST, an anti-rabbit IgG-HRP (1∶3000; 170-6515; Bio-Rad Laboratories, Hercules, CA) was used as secondary antibody. Detection was performed using the Novex ECL HRP chemiluminescent substrate reagent (WP20005; Invitrogen). Further incubation of the membranes with a GAPDH antibody (1∶10000, sc-47724; Santa Cruz Biotechnology Inc.) and an anti-mouse IgG-HRP secondary antibody (1∶10000; sc-2005; Santa Cruz Biotechnology Inc.) were performed for normalization purposes. Signals were visualized on high performance chemiluminescence films (Hyperfilm ECL; GE healthcare) and protein expressions were quantified on the immunoblots using ImageJ version 1.43 u (http://rsb.info.nih.gov/ij/).

### Statistical Analysis

All analyses were performed using MS office Excel 2007, unless otherwise specified. Paired student’s *t*-test was conducted to compare *miR-205* expression in paired clinical samples, and to analyze differences between two experimental groups. Student *t*-test with equal variance was performed to compare mean relative changes between the tested and control samples from three independent experiments. Pearson’s correlation analysis was used to determine the association between *miR-205* and *CYR61* or *CTGF* expression levels. All analyses were 2-tailed and *P*-values <0.05 were considered statistically significant.

## Supporting Information

Figure S1 **Evaluation of** ***miR-205*** **regulation on apoptosis in human cervical cancer cell lines, as evaluated by caspase-3 colorimetric assay.** (A) No significant change of apoptosis was observed in both *miR-205* overexpression and suppression experiments. (B) Positive control for the apoptosis assay. Significant induction of apoptosis was observed in both cell lines after treatment with camptothecin (100 µM) for 15–18 hours. Expression of *miR-205* was significant reduced after treatment with a miRNA inhibitor (C), or increased upon treatment with a miRNA mimic (D). Data represent mean of three independent experiments and error bars indicate standard deviations from the mean. All comparisons were assessed by *t*-test. **P<0.01; ***P<0.001; *n.s.* = not significant.
(PDF)

Figure S2 **Evaluation of Ago2 immunoprecipitation efficiency by qRT-PCR.** Comparisons of *miR-21* (A) and *miR-30a-5p* (B) expression levels before and after immunoprecipitation using anti-Ago2 or anti-IgG isotype control in Pre-miR-205-treated and mock transfected HeLa cells. Error bars indicate standard deviations from the mean of three independent experiments. **P<0.01, *t*-test.
(PDF)

Figure S3 **Clustering analysis of CLIP-Chip** **expression data.** This figure shows six clusters of enriched and depleted genes in *miR-205* overexpression and suppression experiments, respectively. The details of the gene list for each cluster are provided in Table S1.
(PDF)

Figure S4 ***miR-205*** **binding site predictions of** ***CYR61*** **(A) and** ***CTGF*** **(B) by RNAhybrid.**
(PDF)

Table S1 **Expression of Cluster 1–6 gene transcripts enriched at** ***miR-205*** **over-expression or depleted at** ***miR-205*** **suppression.**
(XLS)

Table S2 **Functional annotations of** ***miR-205*** **targets from CLIP-Chip using GENECODIS 2.0.**
(XLS)

Table S3 **Candidate targets from CLIP-Chip experiments were also identified as** ***miR-205*** **targets by computational methods.**
(XLS)
