# Supplementary figures and images for: miR-205 Expression Promotes Cell Proliferation and Migration of Human Cervical Cancer Cells
Source: PLoS One. 2012 Oct 3;7(10):e46990. doi: 10.1371/journal.pone.0046990 (PMC3463520; doi:10.1371/journal.pone.0046990)

**Figure S1**

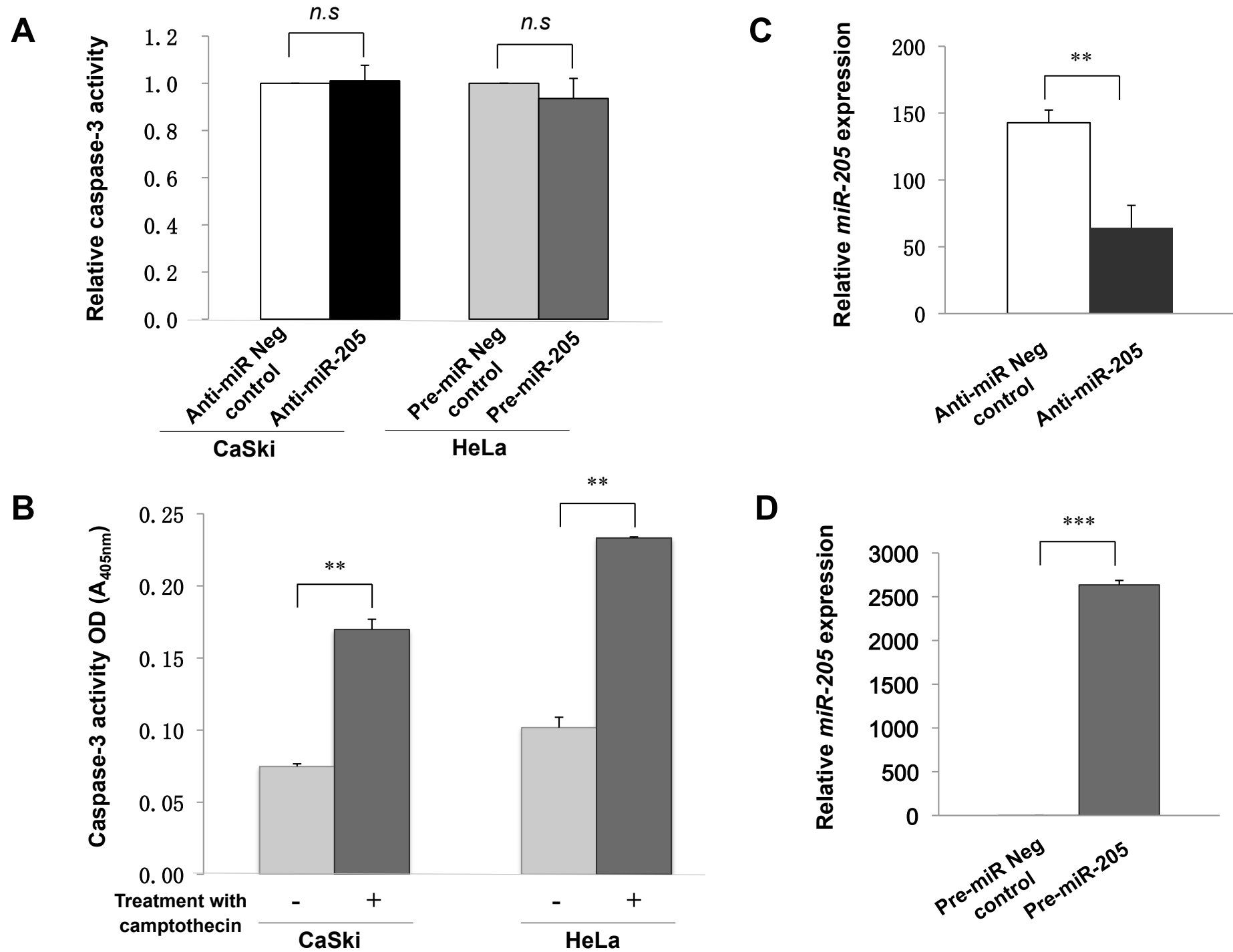

Supplement: Figure S1 — Evaluation of miR-205 regulation on apoptosis in human cervical cancer cell lines, as evaluated by caspase-3 colorimetric assay. (A) No significant change of apoptosis was observed in both miR-205 overexpression and suppression experiments. (B) Positive control for the apoptosis assay. Significant induction of apoptosis was observed in both cell lines after treatment with camptothecin (100 µM) for 15–18 hours. Expression of miR-205 was significant reduced after treatment with a miRNA inhibitor (C), or increased upon treatment with a miRNA mimic (D). Data represent mean of three independent experiments and error bars indicate standard deviations from the mean. All comparisons were assessed by t-test. **P<0.01; ***P<0.001; n.s. = not significant. (PDF) [file pone.0046990.s001.pdf]

Figure S2

A

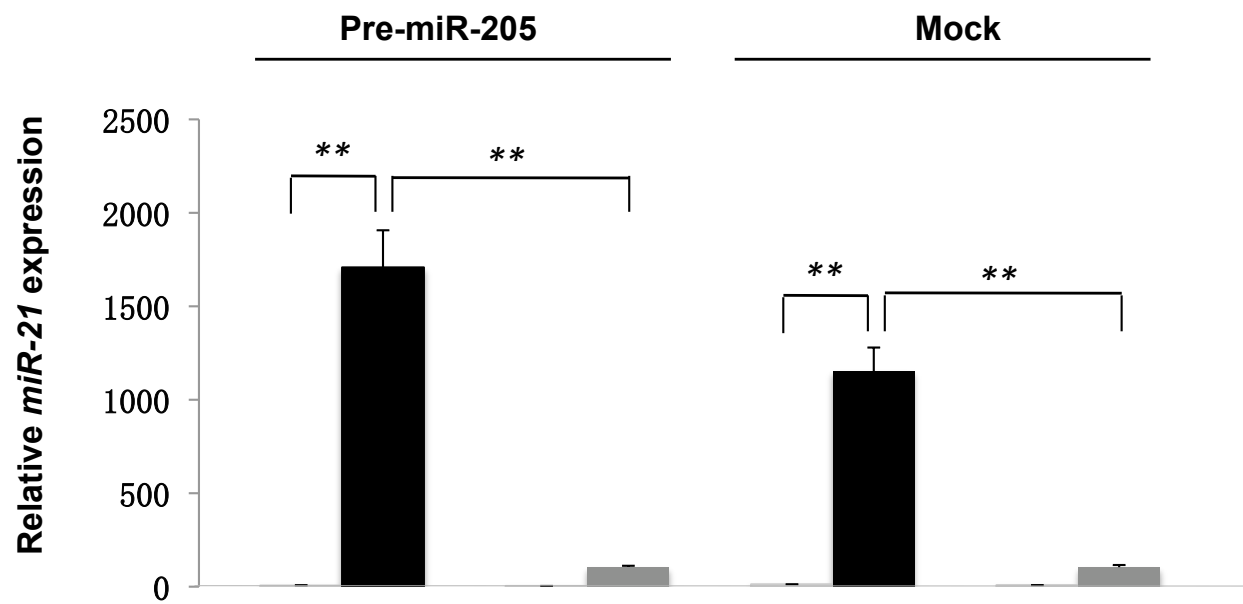

B

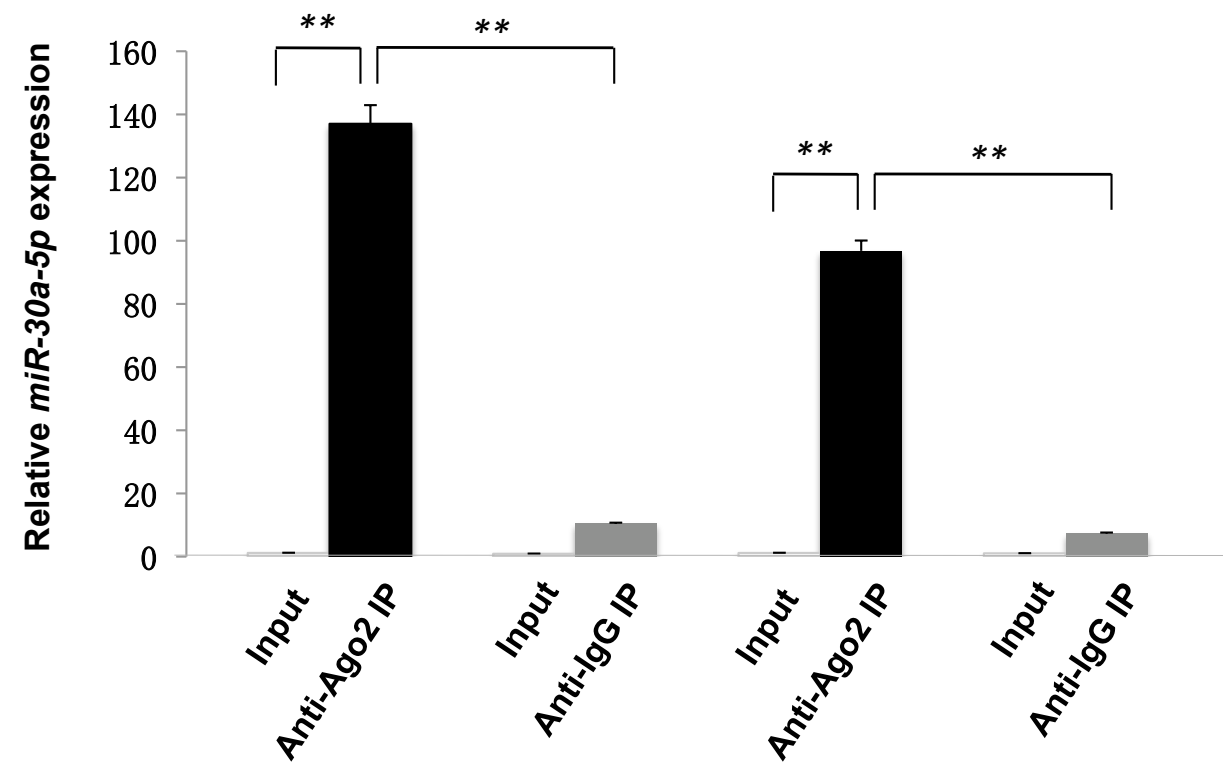

Supplement: Figure S2 — Evaluation of Ago2 immunoprecipitation efficiency by qRT-PCR. Comparisons of miR-21 (A) and miR-30a-5p (B) expression levels before and after immunoprecipitation using anti-Ago2 or anti-IgG isotype control in Pre-miR-205-treated and mock transfected HeLa cells. Error bars indicate standard deviations from the mean of three independent experiments. **P<0.01, t-test. (PDF) [file pone.0046990.s002.pdf]

Figure S3

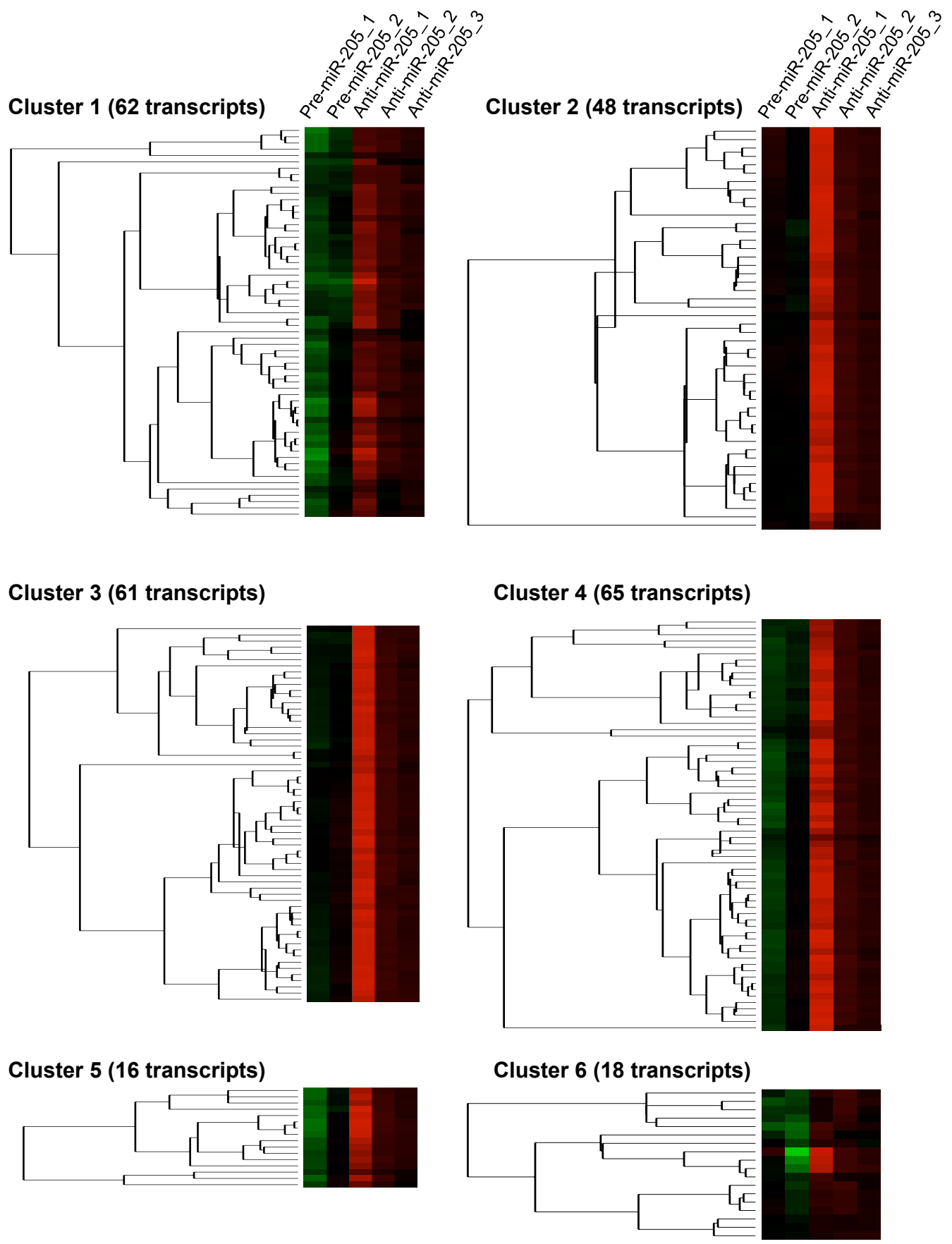

Supplement: Figure S3 — Clustering analysis of CLIP-Chip expression data. This figure shows six clusters of enriched and depleted genes in miR-205 overexpression and suppression experiments, respectively. The details of the gene list for each cluster are provided in Table S1. (PDF) [file pone.0046990.s003.pdf]
