# Supplementary material for: miR-205 Expression Promotes Cell Proliferation and Migration of Human Cervical Cancer Cells
Source: PLoS One. 2012 Oct 3;7(10):e46990. doi: 10.1371/journal.pone.0046990 (PMC3463520; doi:10.1371/journal.pone.0046990)
Supplement: Figure S4 — miR-205 binding site predictions of CYR61 (A) and CTGF (B) by RNAhybrid. (PDF) [file pone.0046990.s004.pdf]

Figure S4

A *CYR61* (NM_001554)

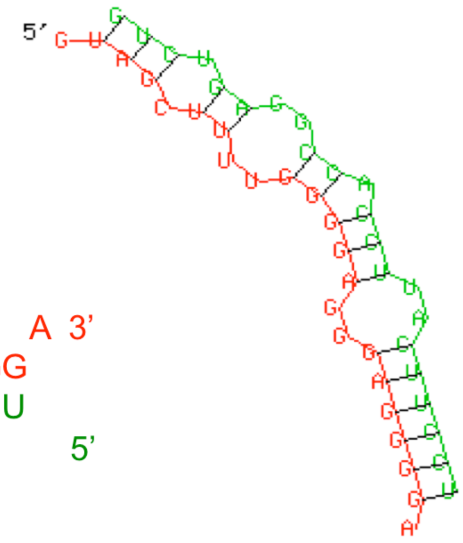

mfe: -26.0 kcal /mol

position: 1911-1933

```
target    5’  G       C      UU             GG              A  3’
                UAG   UU     GG  GGA      GAGGGG
                GUC   GA     CC   CCU      CUUCCU
miR-205   3’          U      GG     A       UA                5’
```

B *CTGF* (NM_001901)

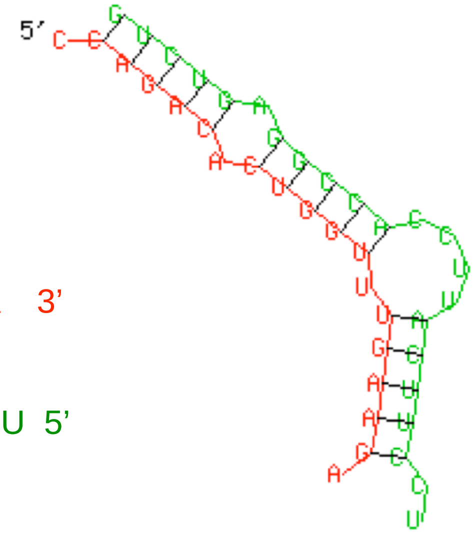

mfe: -22.5 kcal /mol

position: 1435-1453

```
target    5’  C            A            U              A    3’
                CAGAC   CUGGU        UGAAG
                GUCUG  GGCCA         ACUUC
miR-205   3’               A           CCUU            CU  5’
```
